# Sparse Contribution Feature Selection and Classifiers Optimized by Concave-Convex Variation for HCC Image Recognition

**DOI:** 10.1155/2017/9718386

**Published:** 2017-07-17

**Authors:** Wenbo Pang, Huiyan Jiang, Siqi Li

**Affiliations:** Software College, Northeastern University, Shenyang 110819, China

## Abstract

Accurate classification of hepatocellular carcinoma (HCC) image is of great importance in pathology diagnosis and treatment. This paper proposes a concave-convex variation (CCV) method to optimize three classifiers (random forest, support vector machine, and extreme learning machine) for the more accurate HCC image classification results. First, in preprocessing stage, hematoxylin-eosin (H&E) pathological images are enhanced using bilateral filter and each HCC image patch is obtained under the guidance of pathologists. Then, after extracting the complete features of each patch, a new sparse contribution (SC) feature selection model is established to select the beneficial features for each classifier. Finally, a concave-convex variation method is developed to improve the performance of classifiers. Experiments using 1260 HCC image patches demonstrate that our proposed CCV classifiers have improved greatly compared to each original classifier and CCV-random forest (CCV-RF) performs the best for HCC image recognition.

## 1. Introduction

Liver cancer is a malignant tumor with high morbidity, which is a huge challenge that humans must face for a long time. Researches demonstrate that early diagnosis can greatly reduce the incidence of cancer. Computer aided diagnosis is important for early diagnosis, which can largely improve the accuracy of cancer diagnosis. With the rapid development of machine learning, there are many algorithms for image classification, such as *K*-nearest neighbor (*K*NN), support vector machine (SVM), extreme learning machine (ELM), random forests (RF), and artificial neural network (ANN). These methods achieve the considerable results for pathological image classification.

Recently, there are different kinds of classification models for pathological image classification. The paper [1] proposed a novel voting ranking random forests method for HCC image classification. A *k*-nearest neighbor classifier was proposed in [2] based on aggregated distance function, which combined several features (surface description, LBP, heraldic features, and SIFT) together. The paper [3] presented a two-phase hyperspectral image classification method combining Elastic Net Regression and spectral spatial bilateral filtering. The paper [4] created a complete, fully automatic, and efficient clinical decision support system for breast cancer malignancy grading, which made use of both image processing and machine learning techniques to perform the analysis of biopsy slides. The paper [5] used 4 different types of support vector machine classifiers based on the 6 features extracted from the raw gait data, including linear SVM, quadratic SVM, cubic SVM, and Gaussian SVM. A new structural statistical matrix was presented in [6], which was gray level size zone matrix texture descriptor variants. The paper [7] proposed two nuclear- and L2, 1-norm regularized 2D neighborhood preserving projection methods for extracting representative 2D image features. The paper [8] put forward feature importance in nonlinear embedding, an extension of PCA-based feature scoring method to KPCA, as well as several NLDR algorithms that can be cast as variants of KPCA.

However, accurate recognition of cells on pathological images based on hand-crafted features with classifier model remains a challenging task because of two main issues. One is that redundancy features not only influence classification results but also increase computing cost and another is how to optimize classifiers in a new form for more accurate results. To solve these two problems, this paper proposes a concave-convex variation (CCV) method to optimize three classifiers (random forest, support vector machine, and extreme learning machine) for the more accurate HCC image classification results. The main contributions of this paper are as follows. First, for removing the features with redundancy and low contribution value, a new sparse contribution (SC) feature selection method is proposed. Second, CCV model computes weights according to geometrical characteristic of cell nucleus and optimizes three classifiers using the weights.

The remainder of the paper is organized as follows. In Section 2, we introduce the related basic knowledge with respect to this paper. The steps of our proposed model are described in Section 3. Sections 4 and 5 explain the mechanisms of feature selection and CCV, respectively.  Section 6 presents our experimental results and analysis. Finally, conclusions are summarized in Section 7.

## 2. Related Basic Knowledge

### 2.1. Features

Cell image features directly influence the performance of classifier and general divided into three groups, which are intensity, morphology, and texture. Three groups of features are shown in Table 1. Intensity features are mainly obtained by computing the pixel value of the whole image [9]. In the diagnosis of carcinomas, pathologists perform a semiquantitative analysis of a small set of morphological features to determine the cancer's histologic grade [10]. Texture is the most important group of features for classification. There are many applications of texture analysis for image classification [11–13].

### 2.2. Classifiers

(*1) Support Vector Machine*. Support vector machine (SVM) is based on the statistical learning theory, which aims to seek a hyperplane for separating two classification models. Note that our intention of this paper is for the optimization of classifier for recognizing the normal and HCC images. Therefore, we just use original binary SVM to verify our proposed method and parallel hyperplane is used for SVM. In the case of two classes question, given a set of training data (*x*_*i*_, *y*_*i*_), *i* = 1,2,…, *l*, *x* ∈ *R*^*n*^, *y* ∈ {±1}. The hyperplane is described as (*w* · *x*) + *b* = 0, which satisfies the constraint as follows:

(1) min Φw=12w2=12w′·w.

Lagrange function is used to solve the problem of constraint optimization,

(2) max Qa=∑j=1laj−12∑i=1l ∑j=1laiajyiyjxi·xjs.t. ∑j=1lajyj=0,a≥0.

**Figure 1:**
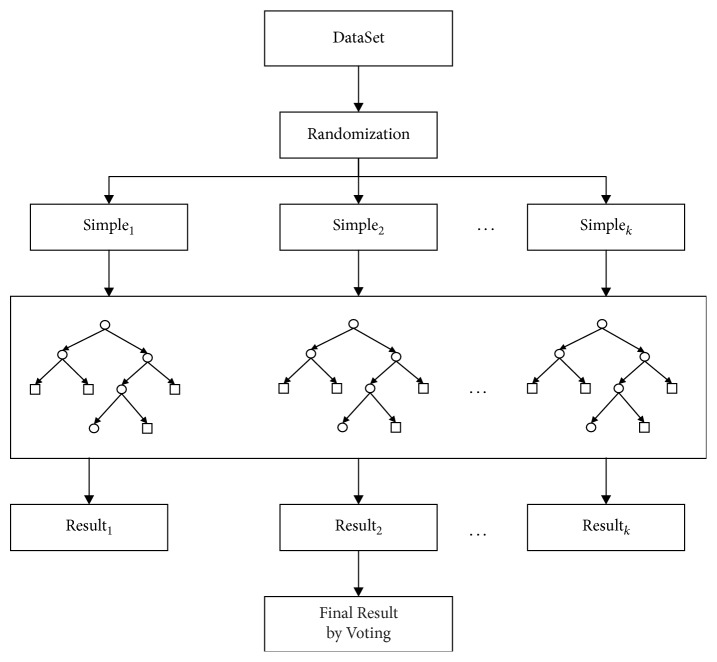
The flow of random forest.

The optimal solution is *a*^*∗*^ = (*a*_1_^*∗*^, *a*_2_^*∗*^,…, *a*_*l*_^*∗*^)^*T*^, where *a* is Lagrange multiplier. Then, the optimal value *w*^*∗*^ and bias *b*^*∗*^ are computed in the following:

(3) w∗=∑j=1laj∗yjxjb∗=y−∑j=1lyjaj∗xj·xj,

where *j* ∈ {*j*∣*a*_*j*_^*∗*^ > 0}. The optimal hyperplane is (*w*^*∗*^ · *x*) + *b*^*∗*^ = 0 and classification function is shown below:

(4) fxsignw∗·x+b∗=sign∑j=1laj∗yjxjxi+b∗,x∈Rn.

(*2) Random Forest*. Random forest (RF) is an ensemble learning method for classification, regression, and other tasks that operate by constructing a multitude of decision trees at training time and outputting the class that is the mode of the classes or mean prediction of the individual trees, which also is a powerful machine learning classifier that is relatively unknown in land remote sensing and has not been evaluated thoroughly by the remote sensing community compared to more conventional pattern recognition techniques [14]. As shown in Figure 1, the flow of RF is described. Firstly, *k* samples are extracted using bootstrap method from the original training set and each sample has the same size. Then, the decision tree models are built for every sample to obtain *k* results of each decision tree. The final result is acquired according to voting of all decision trees.

RF increases the diversity between two classification models by constructing different training sets. After training, there is a sequence of classification models {*h*_1_(*X*), *h*_2_(*X*),…, *h*_*k*_(*X*)}. The final classification results are generated by simple voting based on the sequence. The decision is shown as

(5) $$\begin{array}{c} H\left(\;x\;\right)=arg\;\underset{Y}{max}\overset{k}{\underset{i=1}{\sum }}I\left(\;h_{i}\left(\;x\;\right)=Y\;\right), \end{array}$$

where *H*(*x*) is the model of classification, *h*_*i*_ is single decision-making tree, *Y* is output variable, and *I* is indicative function.

(*3) Extreme Learning Machine*. Extreme learning machine (ELM) is used on image segmentation, classification, and recognition [15, 16]. A brief theory of ELM is given in [17]. A single-hidden-layer feedforward network (SLFN) with *N* arbitrary samples (*x*_*i*_, *t*_*i*_) ∈ *R*^*d*^ × *R*^*m*^ and *L* hidden nodes can be mathematically modeled as follows:

(6) $$\begin{array}{c} \overset{L}{\underset{i=1}{\sum }}\beta_{i}g\left(\;W_{i}\cdot X_{i}+b_{i}\;\right)=o_{j},\;j=1,2,\ldots ,N, \end{array}$$

where *g*(*x*) is activation function, *W*_*i*_ = [*W*_*i*,1_, *W*_*i*,2_,…, *W*_*i*,*n*_]^*T*^ is input weight, *β*_*i*_ is output weight, and *b*_*i*_ is the bias of *i*th hidden layer. SLFN aims to minimize the output error: that is, ∑_*j*=1_^*N*^‖*o*_*j*_ − *t*_*j*_‖ = 0, and *β*_*i*_, *W*_*i*_, and *b*_*i*_ satisfy

(7) $$\begin{array}{c} \overset{L}{\underset{i=1}{\sum }}\beta_{i}g\left(\;W_{i}\cdot X_{i}+b_{i}\;\right)=t_{j},\;j=1,2,\ldots ,N. \end{array}$$

The above *N* equations can be expressed with matrix form,

(8) $$\begin{array}{c} H\beta =T, \end{array}$$

where *H* is output of hidden layer node, *β* is output weight, and *T* is expected output,

(9) H=gW1·X1+b1⋯gWL·X1+bL⋮⋱⋮gW1·XN+b1⋯gWL·XN+bLN×Lβ=β1T⋮βLTL×m,T=T1T⋮TNTN×m.

For training SLFN, ${\hat{W}}_{i}$, ${\hat{b}}_{i}$, and ${\hat{\beta }}_{i}$ are described,

(10) $$\begin{array}{c} \left\|\;H\left(\;{\hat{W}}_{i},{\hat{b}}_{i}\;\right){\hat{\beta }}_{i}-T\;\right\|=\underset{W,b,\beta }{min}\left\|\;H\left(\;W_{i},b_{i}\;\right)\beta_{i}-T\;\right\|, \end{array}$$

where *i* = 1,2,…, *L*. After randomly generating the hidden node parameters, training an SLFN is equivalent to find a least-squares solution of *Hβ* = *T* and *β* can be decided by

(11) $$\begin{array}{c} \hat{\beta }=H^{†}T, \end{array}$$

where *H*^†^ is the Moore-Penrose generalized inverse of *H*.

### 2.3. Hematoxylin and Eosin Pathological Image

Hematoxylin and eosin (H&E) staining is ubiquitous in pathology practice and research, which facilitates pathologist interpretation of microscopic slides by enhancing the contrast between cell nuclei and other histological structures. This allows pathologists to visually identify cellular components, extracellular structures, and lumen with relative ease [18]. This paper thus uses H&E pathological images as experiment data set and each pathological image was selected by pathologists.

## 3. Classification Model and Steps

**Figure 2:**
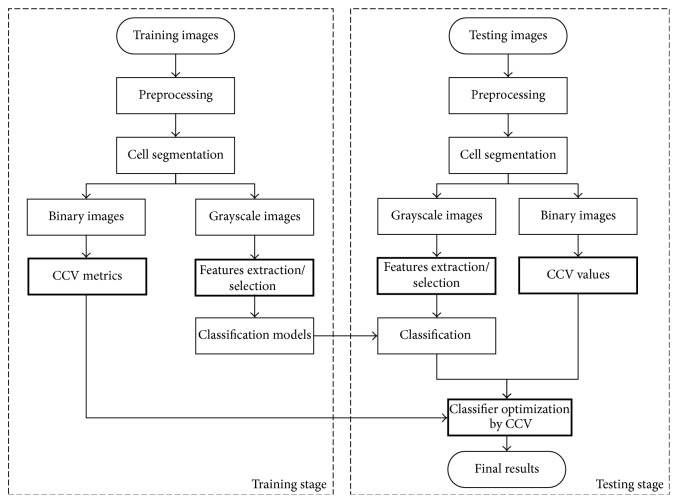
Our proposed classification model flowchart.

As shown in Figure 2, our proposed classification model is divided into two phases, training and testing.

In training stage, the whole slide images (WSI) are preprocessed using bilateral filtering firstly. Then, normal and HCC cells are selected from WSI under the guidance of pathologist. Then, single cell images are obtained by cell segmentation with a size of 64 × 64. Next, this paper extracts three groups gray features on gray-scale images, which are intensity, morphology, and texture features. A sparse contribution (SC) feature selection model is developed to select features. Subsequently, three classifiers are trained using the selected features. Meanwhile, the concave-convex variation (CCV) model is established through the calculation of each binary image CCV, which will optimize three original classifiers in the testing.

During testing stage, similarly, the testing images are preprocessed with bilateral filtering. Next, gray-scale and binary images involving the segmented cells are obtained. Testing images extract and select features as with training stage. After that, initial classification results are acquired through the trained original classifiers. For our contribution of this paper, the more accurate result is calculated using the optimized classifier with CCV model.

## 4. Feature Extraction and Selection

### 4.1. Feature Extraction

Feature extraction is an important step of image classification. As described in Section 2.1, this paper extracts three groups of features. For the computation of the intensity features, we compute every single pixel's gray value of the whole images. This group can represent the global feature of each whole image. In addition, this paper regards density, nucleocytoplasmic ratio as morphology features. The greatest contribution group in this paper is texture feature and the specific steps of texture features are explained in the following.

(*1) Gray Level Cooccurrence Matrix*. Gray Level Cooccurrence Matrix (GLCM) was one of the earliest techniques used for image texture analysis [19]. Several typical parameters of GLCM are used to represent image texture condition. In GLCM, angular second moment (ASM) is used to reflect image gray distribution uniformity and textural detail; contrast (CON) can reflect the comparison between image pixel values; entropy (ENT) reflects the image gray distribution complexity and heterogeneity; correlation (COR) is used to reflect local gray correlation in image; inverse different moment (IDM) reflects the homogeneity of image texture. The computational formulas of these five parameters are shown in

(12) ASM=∑i=1N ∑j=1NIi,j2ENT=−∑iN∑jNIi,jln⁡Ii,jCON=∑iN∑jNi−j2Ii,jCOR=∑iN∑jNi,jIi,j−uxuyσxσyIDM=∑iN∑jNIi,j1+i+j2,

where *I*(*i*, *j*) is the element of the *i*th row and *j*th column.

(*2) Local Binary Pattern*. Local binary pattern (LBP) was utilized to describe the local texture feature of images proposed by Zhao et al. [20]. LBP has the characters of rotational invariance and gray-scale invariance. The steps of LBP in this paper are shown as follows:

- Divide the input image into cells with size of 16 × 16.
- For every pixel in each cell, compare its gray value with neighboring 8 pixels. If the center pixel's gray value is less than the neighboring pixel's gray value, then mark the neighboring pixel as 1; otherwise, mark it as 0. Each 3 × 3 cell can generate an 8-bit binary code, which can be used as LBP value of cell's center pixel.
- Plot each cell's histogram with computing the probability of each number (assume it as decimal number) and then normalize the histogram.
- Obtain the LBP textural feature of whole image by connecting each cell's histogram into a feature vector.

(*3) Scale-Invariant Feature Transform*. Scale-invariant feature transform (SIFT) is an image descriptor for image-based matching and recognition developed by Lindeberg [21], which is invariance on rotation, measure, and angle of view. The first step of SIFT is to build scale space, which uses Gaussian convolution kernel as linear kernel. The definitions of the scale space and Gaussian function are shown in

(13) Lx,y,σ=Gx,y,σIx,yGx,y,σ=12πσ2e−x2+y2/2σ2.

When the building of scale space is completed, SIFT detects and locates the extreme point of the scale space. Then, assign direction parameters for each key point and the definitions of these parameters are shown in

(14) valx,y=Lx+1,y−Lx−1,y2+Lx,y+1−Lx,y−12dirx,y=a tan2⁡Lx,y+1−Lx,y−1Lx+1,y−Lx−1,y.

The last step of SIFT is to generate the descriptor of key points.

(*4) Tamura*. Tamura features include contrast, coarseness, directionality, line-likeness, regularity, and roughness, where the contrast, coarseness, and directionality are more important in image identification. This paper uses former three features and their definitions and computation methods are given by [22].

Following these methods of feature extraction, this paper extracts 463 dimensions' features for each patch, including intensity, morphology, and texture.  Table 2 presents the number of each class's features.

### 4.2. Sparse Contribution Feature Selection Model

After obtaining the complete features of HCC, feature selection is a normal practice. In recent years, some methods [23–26] are proposed for feature selection, such as filter, wrapper, and embedded. Feature selection aims to find out a beneficial subset from original feature, which can contribute more for final recognition. The purposes of feature selection are thus summarized as follows: (1) increasing the accuracy of prediction, (2) building a fast and efficient model, and (3) being applicable more to recognition model. Following these purposes, a new sparse contribution (SC) feature selection model is developed to select a beneficial feature subset for HCC image classification. The concrete procedures are as follows.

**Step 1 .**

Let *fs* denote the complete HCC image feature set, which is formulated as

(15) $$\begin{array}{c} \text{fs}={\left(\;\begin{array}{ccc} f_{11} & \cdots & f_{1n}\\ \vdots & \ddots & \vdots \\ f_{m1} & \cdots & f_{mn} \end{array}\;\right)}_{m\times n}, \end{array}$$

where *m* is the number of the extracted features and *n* is the number of the training samples. *f*_*ij*_ represents the value of *i*th feature of the *j*th sample. First, the feature data of each row is normalized from 0 to 1. Nest, according to Pearson coefficient, we calculate the correlation coefficient between each two characteristics. The absolute value of correlation coefficient is close to 1, and the redundancy is strong. Finally, based on this rule, the redundant features are removed.

**Step 2 .**

**Figure 3:**
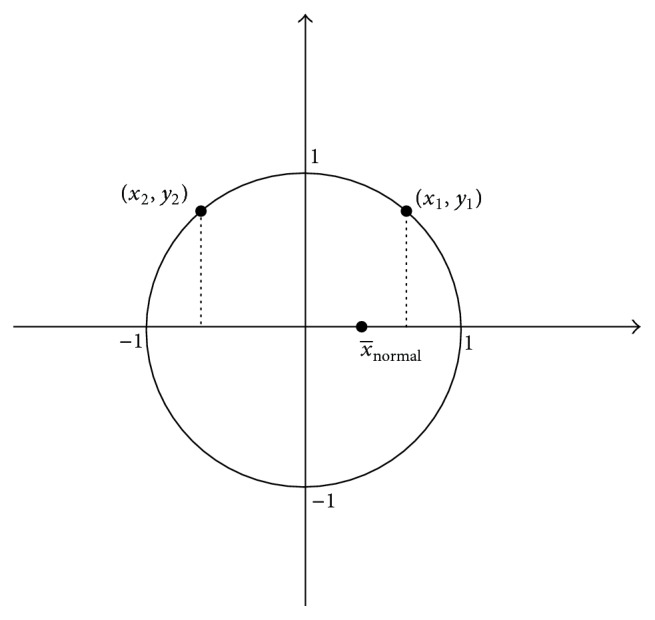
An example of the calculation of contribution value.

After normalizing the data, a contrast mapping is performed to normalize the feature data of each row from −1 to 1. It could be calculated through the following formula:

(16) $$\begin{array}{c} x_{*}=\frac{x_{i}-\mu }{\sqrt{\sigma }+\varepsilon }, \end{array}$$

where *μ* and $\sqrt{\sigma }$ represent the average value and standard deviation of each row, respectively. *x*_*i*_ is a feature value and *i* = 1,2,…, *n*. *ε* is a control parameter, and for each row of fs, a suitable *ε* is selected to satisfy *x*_*∗*_ range from −1 to 1 and obey approximately normal distribution. The updated fs is thus represented as follows:

(17) $$\begin{array}{c} fs_{1}={\left(\;\begin{array}{ccc} x_{11} & \cdots & x_{1n}\\ \vdots & \ddots & \vdots \\ x_{m_{1}1} & \cdots & x_{m_{1}n} \end{array}\;\right)}_{m_{1}\times n}, \end{array}$$

where *x* represents the normalized data from −1 to 1. fs_1_ is selected from (15), where *m*_1_ < *m*. Next, we design a unit circle mapping to calculate the contribution value of each feature. For the sake of notational simplicity, let (*x*_1_, *x*_2_,…, *x*_*n*_) be a row of fs_1_ that represents one feature of all training sample. All training sample is divided into two categories, normal and abnormal. ${\overset{-}{x}}_{normal}$ and ${\overset{-}{x}}_{abnormal}$ represent the average values of (*x*_1_, *x*_2_,…, *x*_*n*_) according to training labels. If ${\overset{-}{x}}_{normal}\cdot {\overset{-}{x}}_{abnormal}\geq 0$, the feature is removed. In another case, the contribution value of each feature is obtained through a unit circle mapping.  Figure 3 shows an example of the calculation of contribution value. If ${\overset{-}{x}}_{normal}>0$, the normal data where *x*_*i*_ > 0 has positive contribution value for HCC image classification. On the contrary, the normal data where *x*_*i*_ < 0 has negative contribution value. *x*_1_ is a normal data and *y*_1_ is the corresponding vertical coordinate of a unit circle, and (1 − *y*_1_) represents the contribution value of *x*_1_. *x*_2_ is also normal data but *x*_2_ < 0, −(1 − *y*_2_) represents the contribution value of *x*_2_. The contribution value of each feature (*C*(*x*)) is acquired summing the contribution values of normal data (*C*(*x*_normal_)) and abnormal data (*C*(*x*_abnormal_)). A threshold *δ*_1_ is selected that if the contribution value of one feature is less than *δ*_1_, the feature is removed.

**Step 3 .**

Based on the first two steps, a new feature set is represented as follows:

(18) $$\begin{array}{c} fs_{2}={\left(\;\begin{array}{ccc} x_{11} & \cdots & x_{1n}\\ \vdots & \ddots & \vdots \\ x_{m_{2}1} & \cdots & x_{m_{2}n} \end{array}\;\right)}_{m_{2}\times n}, \end{array}$$

where *x*_*m*_2_*n*_ represents the selected features of all training data. To improve computational efficiency, *x*_*ij*_ in fs_2_, which are closed to 0 (|*x*_*ij*_| ≤ *δ*_2_), are set to 0. Finally, feature set of the training HCC images is expressed in the following:

(19) $$\begin{array}{c} fs_{final}={\left(\;\begin{array}{cccc} x_{11} & 0 & \cdots & x_{1n}\\ x_{21} & 0 & \cdots & x_{2n}\\ \cdot & \cdot & & \cdot \\ x_{k1} & 0 & \cdots & x_{kn} \end{array}\;\right)}_{k\times n}, \end{array}$$

where *k* = *m*_2_ < *m*_1_ < *m* and it is seen that fs_final_ is a sparse feature matrix.

In the proposed feature selection method, there are three important characteristics with respect to HCC image feature set. First, fs_final_ removes the features with redundancy and low contribution value. Second, fs_final_ remains the approximate uniform of all features distribution. Third, for a more straightforward training model, *δ*_2_ is utilized to satisfy fs_final_ lifetime sparsity [27]. Beside the selected feature set, the optimized classifiers influence classification performance significantly, which will be investigated in the next section.

## 5. Classifiers Optimized with Concave-Convex Variation

Concavity-convexity is an essential feature for image process [28]. The contour of a region is defined as convexity if the line between two random points of the region is still located in the region; otherwise, it is defined as concavity. For HCC images, according to statistics and a priori knowledge of pathologist, the outline of normal nucleus could be regarded as convexity approximately, which is more regular than abnormal nucleus.

 Following this a priori knowledge, different from the concave-convex feature in [28], concave-convex variation (CCV) is proposed to optimize three classifiers (RF, SVM, and ELM) in this paper. The inflection points of a curve are calculated through *f*′′(*x*) = 0, where *f*(*x*) is the analytic expression of the curve. However, in general, *f*(*x*) is uncharted because of the irregularity of each nucleus contour. To address this problem, the CCV models of normal nucleus and abnormal nucleus are established using a slope approximation method to optimize the classifiers for more accurate accuracy. The specific steps are as follows.

**Step 1 .**

A circumscribed rectangle of the contour is built through four horizontal and vertical lines. In this way, 4 tangent points are acquired, which are marked as (*x*_left_, *y*_left_), (*x*_right_, *y*_right_), (*x*_top_, *y*_top_), and (*x*_bottom_, *y*_bottom_). The contour is divided into 4 curves by these 4 tangent points, which can be efficiently utilized in the next step.

**Step 2 .**

**Figure 4:**
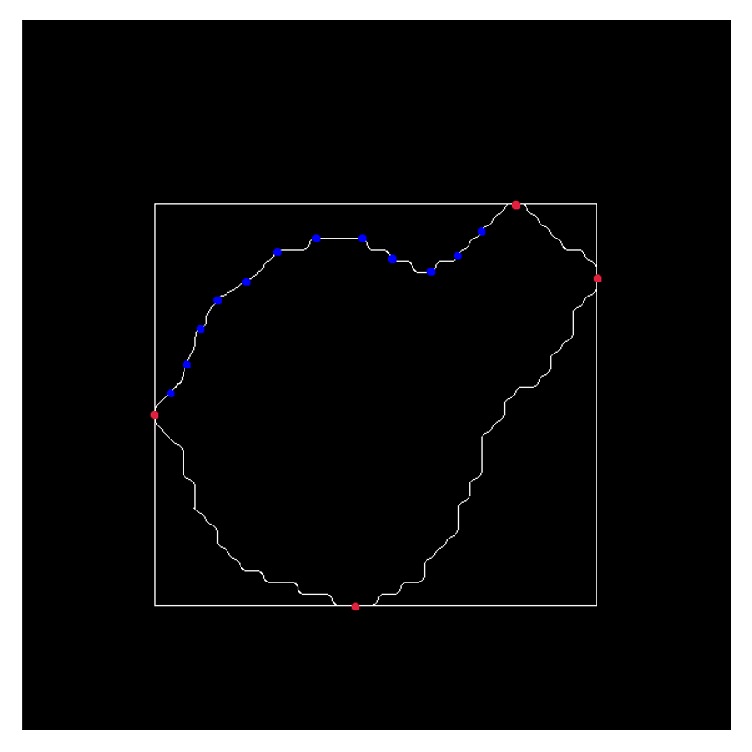
The edge curve and exterior rectangle of cell. The red points represent tangent points and blue points express select points for computing slope vector.

As shown in Figure 4, for a curve, we get the points on the curve with 2 pixel intervals between (*x*_left_, *y*_left_) and (*x*_top_, *y*_top_) and mark them as (*x*_1_, *y*_1_), (*x*_2_, *y*_2_),…, (*x*_*i*_, *y*_*i*_). Then, we compute the slopes between (*x*_left_, *y*_left_) and the other all selected points through the following formula:

(20) $$\begin{array}{c} k=\frac{y_{1}-y_{2}}{x_{1}-x_{2}}. \end{array}$$

A slope vector, *K* = [*k*_1,left_, *k*_2,left_,…, *k*_*i*,left_, *k*_top,left_], is constructed using (5).

**Step 3 .**

For writing convenience, let *K* = [*k*_1,left_, *k*_2,left_,…, *k*_*i*,left_, *k*_top,left_] represent *K* = [*k*_1_, *k*_2_,…, *k*_*i*_, *k*_*i*+1_]. The slope-difference vector, Δ*K* = [Δ*k*_1_, Δ*k*_2_,…, Δ*k*_*i*_], is formed using Δ*k*_*n*_ = *k*_*n*+1_ − *k*_*n*_, *n* = 1,2,…, *i*. We record the number of symbol opposites between each two adjacent Δ*k*_*i*_. The number is considered as CCV(*c*), where *c* represents a nucleus contour. In other words, if a Δ*K* = [+, +, −, −, +, −], the CCV number is 3. This is the slope approximation method to describe the CCV of each nucleus and the CCV classifier is detailed as explained in the next steps.

**Step 4 .**

After obtaining each HCC image's CCV, the CCV models of normal and abnormal are established according to the labels of all training data. mean(CCV_normal_) and mean(CCV_abnormal_) are defined as the mean CCV value of normal data and abnormal data, respectively.

**Step 5 .**

A classification result of each original classifier (RF, SVM, and ELM) without CCV is acquired and it can provide the probability of each nucleus belonging to normal or abnormal. All testing data is roughly divided into two parts. For the classified normal testing data, CCV_testing_(*c*_normal_) represents CCV of each classified testing normal data, and the adjusting weight *ω*_normal_ is set as

(21) $$\begin{array}{c} \omega_{normal}=\left\{\begin{array}{cc} 1.2, & if\;\;{CCV}_{testing}\left(c_{normal}\right)\leq m_{1}\\ 1.2+\frac{0.4\left({CCV}_{testing}\left(c_{normal}\right)-m_{1}\right)}{m_{1}-m_{2}} & \text{if }m_{1}<{CCV}_{testing}\left(c_{normal}\right)\leq \frac{m_{1}+m_{2}}{2}\\ 0.8+\frac{0.4\left({CCV}_{testing}\left(c_{normal}\right)-m_{2}\right)}{m_{1}-m_{2}} & \text{if }\frac{m_{1}+m_{2}}{2}<{CCV}_{testing}\left(c_{normal}\right)<m_{2}\\ 0.8, & if\;\;{CCV}_{testing}\left(c_{normal}\right)\leq m_{2}, \end{array}\right. \end{array}$$

where *m*_1_ = mean(CCV_normal_) and *m*_2_ = mean(CCV_abnormal_), the updated probability of each testing normal data, and *p*_normal_^final^ = *p*_normal_^initial^ · *ω*_normal_, where *p*_normal_^initial^ represents the initial probability of each classified normal data. If *p*_normal_^final^ ≥ 1/2, the initial classified normal HCC images are labeled as normal; otherwise, they are abnormal. For the classified abnormal testing data, the adjusting weight *ω*_abnormal_ is defined as

(22) $$\begin{array}{c} \omega_{abnormal}=\left\{\begin{array}{cc} 1.2, & if\;\;{CCV}_{testing}\left(c_{abnormal}\right)\leq m_{2}\\ 1.2+\frac{0.4\left({CCV}_{testing}\left(c_{abnormal}\right)-m_{2}\right)}{m_{2}-m_{1}} & \text{if }\frac{m_{1}+m_{2}}{2}<{CCV}_{testing}\left(c_{abnormal}\right)\leq m_{2}\\ 0.8+\frac{0.4\left({CCV}_{testing}\left(c_{abnormal}\right)-m_{1}\right)}{m_{2}-m_{1}} & \text{if }m_{1}<{CCV}_{testing}\left(c_{abnormal}\right)<\frac{m_{1}+m_{2}}{2}\\ 0.8, & if\;\;{CCV}_{testing}\left(c_{abnormal}\right)\leq m_{1}, \end{array}\right. \end{array}$$

where CCV_testing_(*c*_abnormal_) is the CCV of each initial classified testing abnormal data. Similarly, if *p*_abnormal_^final^ = *p*_abnormal_^initial^.*ω*_abnormal_ ≥ 1/2, the initial classified abnormal HCC images are labeled as abnormal; otherwise, they are normal.

Following this method, the label of each HCC testing image is obtained using optimized CCV classifiers. To verify the effectiveness of CCV, three common classifiers (RF, SVM, and ELM) are utilized and the experiment results demonstrate that our proposed CCV classifiers improve classification performance than the corresponding original classifiers.

## 6. Results and Discussions

### 6.1. Experimental Platform and Data

The experimental platform is Intel(R) Core(TM) i7-4790 CPU@3.60 GHz, 8 G RAM, 930 G hard disk, Windows 7 OS, and MATLAB R2016a simulation environment.

**Figure 5:**
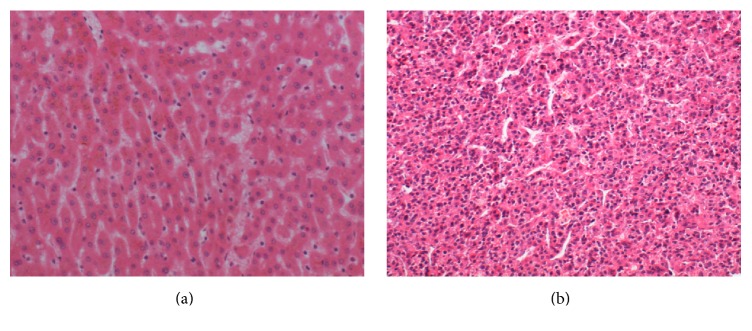
The liver tissue pathology images. (a) is normal image and (b) shows HCC image.

The Experimental Data of this paper are obtained by the pathology department of a large hospital in Shenyang, China. 96 hematoxylin-eosin (H&E) pathological images are used in our experiment. The picture format is TIFF and spatial resolution is 1280 × 960. The magnification of pathology images is 400.  Figure 5 presents the liver tissue pathology images; Figures 5(a) and 5(b) show a normal and HCC image, respectively.

**Figure 6:**
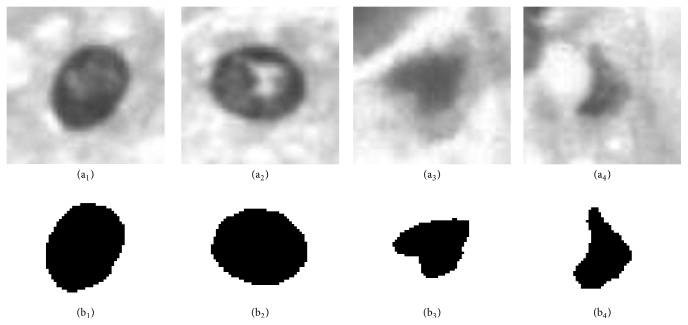
The cell gray-scale images and the cell binary images. (a_1_, a_2_) are normal cell gray-scale image and (b_1_, b_2_) are the matching binary images. (a_3_, a_4_) are HCC cell gray-scale image and (b_3_, b_4_) are the matching binary images.

As mentioned in Section 3, this paper segments the WSI to obtain the single cell images. The cell gray-scale images and the cell binary images are shown in Figure 6. The number of training images and testing images is shown in Table 3.

### 6.2. Experimental Results

#### 6.2.1. Experimental Evaluative Criteria

This paper uses accuracy (ACC), sensitivity (SEN), and specificity (SPE) to evaluate the performance of classification. Sensitivity is the proportion of HCC cell images that are correctly classified and specificity indicates the rate of normal cell images that are correctly classified. *F*1-Score also is used for comparing the performance of classifiers before and after optimization. The definitions of the evaluation criterion are shown in

(23) ACC=TP+TNTP+FN+TN+FPSEN=TPTP+FNSPE=TNTN+FPP=TPTP+FPR=TPTP+FNF1=2PRP+R,

where TP and TN are the correctly classified cell images in total images. FP and FN are the wrongly classified cell images.

#### 6.2.2. Performance Comparisons

**Figure 7:**
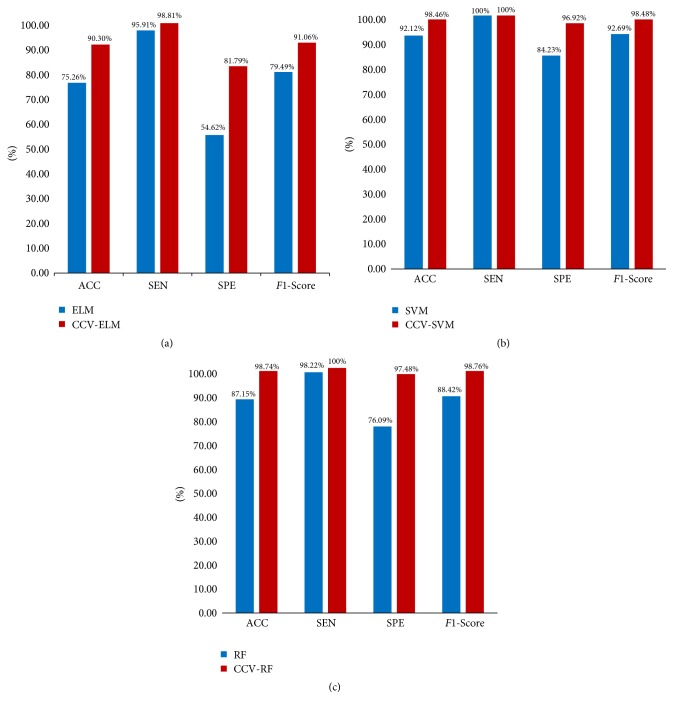
The performance comparison of three classifiers. (a) ELM versus CCV-ELM, (b) SVM versus CCV-SVM, and (c) RF versus CCV-RF. The blue bars represent original classifiers and the red bars are the classifiers optimized by CCV.

As introduced in Section 5, CCV can apply to multiple classifiers. Thus, RF, SVM, and ELM are used in this paper. The comparison results between CCV and original classifiers are shown in Figure 7; Figure 7(a) describes ELM versus CCV-ELM.  Figure 7(b) presents SVM versus CCV-SVM and Figure 7(c) shows RF versus CCV-RF.

Based on Figure 7, we can see that CCV-based classifiers all enhance performance compared to the original classifiers in terms of ACC, SEN, SPE, and *F*1-Score. For ELM, the classification accuracy increases from 75.26% to 90.30% and specificity could achieve 81.79%. With regard to SVM, it performs best in three classifiers without optimizing by CCV. CCV could still further boost the ACC from 92.12% to 98.46%. Finally, RF performs better than ELM and poorer than SVM. However, CCV-based RF could achieve the best performance among these three classifiers with 98.74% classification accuracy.

This paper also plots precision and recall (*P*-*R*) curve and receiver operating characteristic (ROC) curve for the further testing of CCV optimization ability. *P*-*R* curve is plotted with precision and recall, which can indicate the classifier performance, visually. ROC curve is plotted with true positive rate (TPR) and false positive rate. The computational formulas of TPR and FPR are shown in

(24) TPR=TPTP+FNFPR=FPTN+FP.

**Figure 8:**
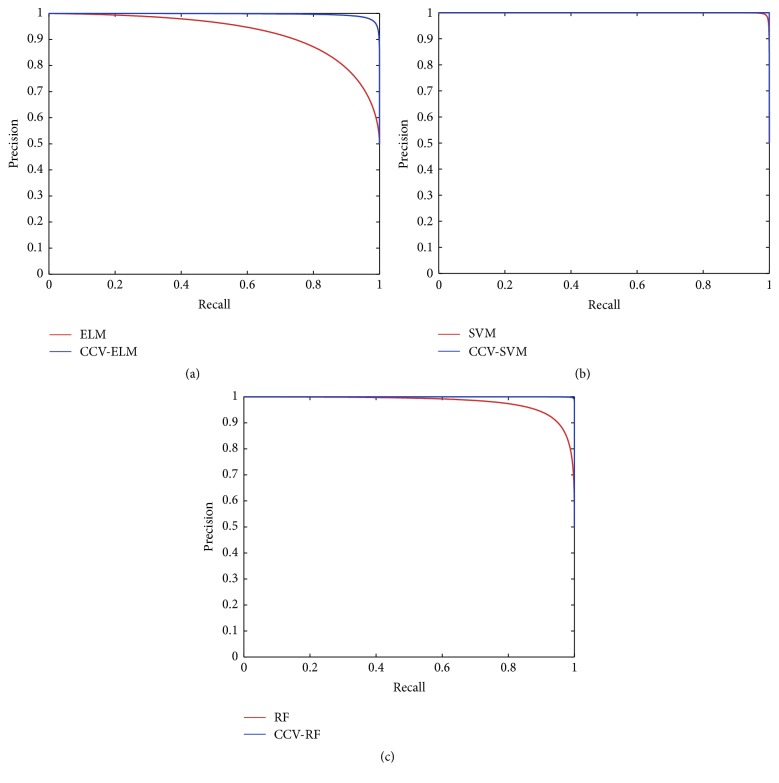
*P*-*R* curves of three classifiers. (a) ELM versus CCV-ELM, (b) SVM versus CCV-SVM, and (c) RF versus CCV-RF. The red curves represent original classifiers and the blue curves are the classifiers optimized by CCV.

**Figure 9:**
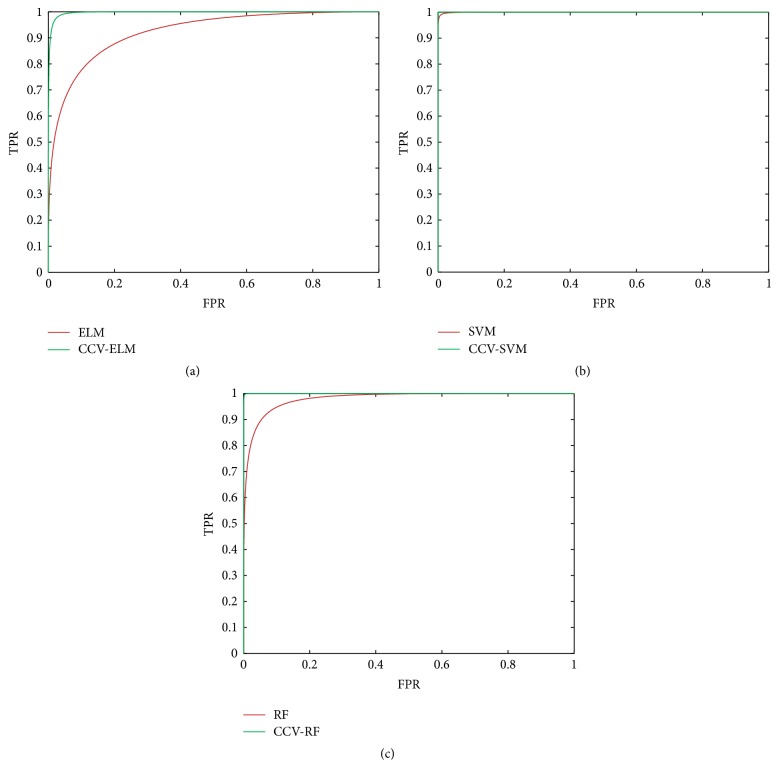
ROC curves of three classifiers. (a) ELM versus CCV-ELM, (b) SVM versus CCV-SVM, and (c) RF versus CCV-RF. The red curves represent original classifiers and the green curves are the classifiers optimized by CCV.

Figures 8 and 9 show *P*-*R* curves and ROC curves of classifiers with and without CCV, respectively. Similarly, classifiers optimized by CCV perform better than original.

Figure 8 shows the *P*-*R* curves of three classifiers with and without CCV, where the red curves represent original classifiers and the blue curves are the classifiers optimized by CCV. As shown in Figure 8(a), CCV is significantly effective to ELM.  Figure 8(b) shows that SVM performs well before and after optimizing by CCV.  Figure 8(c) is the comparison of RF; we can see that RF performs deficiently without CCV and performs the best after optimization.  Figure 9 exhibits the ROC curves of three classifiers. The red curves represent original classifiers and the green curves are the classifiers optimized by CCV. We can obtain the same conclusions with Figure 8. To sum up, CCV can improve performance of three classifiers, in which the CCV-RF for HCC image classification performs best in our experiments.

#### 6.2.3. Comparison with Other Works

**Figure 10:**
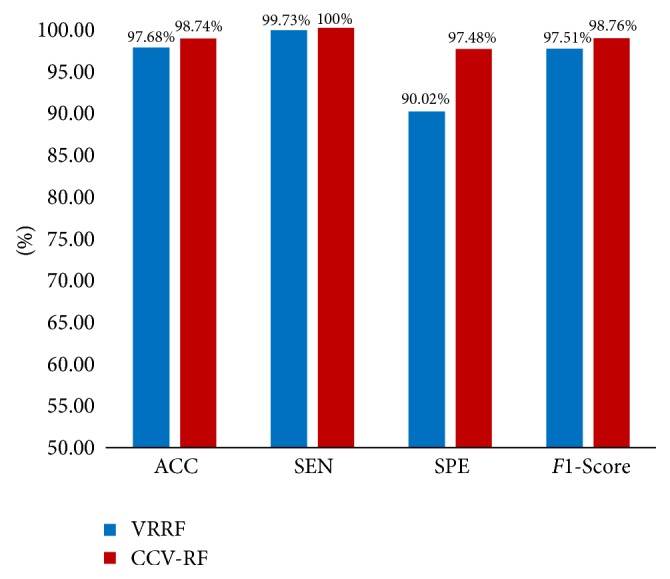
The performance comparison of VRRF and CCV-RF. The blue bars represent VRRF and the red bars show the RF optimized by CCV.

[1] proposed a novel voting ranking random forests (VRRF) method to solve HCC image classification problem, which is based on conventional random forests model and optimized the voting step later. This paper compares the performance between VRRF and CCV-RF in terms of ACC, SEN SPE, and *F*1-Score, which use the same data set in our paper. The comparison result is shown in Figure 10.

According to Figure 10, we can see that both VRRF and CCV-RF perform well on HCC image classification. The classification accuracy of 97.68% and 98.74% can be achieved using VRRF and CCV-RF, respectively. SEN and *F*1-Score values of VRRF are also close to CCV-RF. However, CCV-RF perform better than VRRF in terms of SPE, which can achieve 97.48%. In addition, different from VRRF, our proposed CCV method could apply to various classifiers.

#### 6.2.4. Feature Selection Performance

In Section 4.2, SC feature selection model is developed for more efficient performances, which can not only improve the accuracy of classification but also reduce the computing cost. In our experiments, the number of feature dimension is reduced from 463 to 89. What is more, to further improve computational efficiency, some elements of matrix are set to 0 for sparse feature matrix.  Table 4 shows the running time of three classifiers before and after feature selection.  Table 5 presents the accuracy of three classifiers without CCV.

Following Tables 4 and 5, it is seen that the running time of each classifier is reduced and the ACC are all increased. After feature selection, the selected beneficial features improve the accuracy and meanwhile reduce the running time. In addition, the construction of sparse feature matrix is also a significant factor for reducing computing time.

### 6.3. Discussions

According to compared results and analysis above, our proposed classifiers optimized by CCV improve classification performance compared to original. Different from concavity-convexity feature, the CCV model utilizes statistical property and difference between the testing CCV and the training mean CCV to adjust the optimized weights. The rule of adjusting weight is based on the Euclidean distance of CCV difference model. In addition, instead of parameter optimization for each classifier, the CCV model considers the difference among samples rather than the performance of classifiers.

## 7. Conclusions

This paper proposes a concave-convex variation (CCV) method to optimize three classifiers (random forest, support vector machine, and extreme learning machine). A new SC feature selection method is developed to remove redundancy features from complete feature set and it is beneficial for final classification and reducing the computational cost. Each classifier provides initial classification results and the corresponding probability. Then, we establish CCV statistical model according to all training data. The final classification results are obtained through CCV classifiers using the rule of adjusting weight. Experiments with 1260 HCC image patches demonstrate that our proposed CCV classifiers perform better than original classifiers in terms of ACC, SEN SPE, and *F*1-Score. Furthermore, the CCV-RF for HCC image classification performs best in this paper.

## Figures and Tables

**Table 1:** Nuclei feature used in histopathology.

Category	Features
Intensity	Density, mean, median, variance, kurtosis, skewness, and so on
Morphology	Area, perimeter, diameter, area overlap ratio, center of mass, minor axis, major axis, smoothness, symmetry, and so on
Texture	Gray level cooccurrence matrix, local binary pattern, scale-invariant feature transform Tamura, fractal, Markov random field, wavelets, Haar-like features, Gabor, run-length, and so on

**Table 2:** The number of each class's features.

Intensity features	Morphology features	Texture features	Total features
21	185	257	463

**Table 3:** The number of training images and testing images used in experiment.

Image type	Training images	Testing images	Total images
Normal images	441	189	630
HCC images	441	189	630
Total images	882	378	1260

**Table 4:** The running time of three classifiers.

	ELM	SVM	RF
Before selection	2.320221 s	463.804518 s	35.183513 s
After selection	1.900448 s	262.635644 s	18.348838 s

**Table 5:** The ACC of three classifiers.

	ELM	SVM	RF
Before selection	63.14%	85.97%	78.62%
After selection	75.26%	92.12%	87.15%

## References

[1] Xia B., Jiang H., Liu H., Yi D. (2016). A novel hepatocellular carcinoma image classification method based on voting ranking random forests. *Computational and Mathematical Methods in Medicine*.

[2] Stoklasa R., Majtner T., Svoboda D. (2014). Efficient *k*-NN based HEp-2 cells classifier. *Pattern Recognition*.

[3] Soomro S. H., Xiao L., Soomro B. N. Hyperspectral image classification via Elastic Net Regression and bilateral filtering.

[4] Krawczyk B., Galar M., Jeleń Ł., Herrera F. (2016). Evolutionary undersampling boosting for imbalanced classification of breast cancer malignancy. *Applied Soft Computing Journal*.

[5] Nakano T., Nukala B. T., Zupancic S. Gaits classification of normal vs. patients by wireless gait sensor and Support Vector Machine (SVM) classifier.

[6] Thibault G., Angulo J., Meyer F. (2014). Advanced statistical matrices for texture characterization: application to cell classification. *IEEE Transactions on Biomedical Engineering*.

[7] Zhang Z., Li F., Zhao M., Zhang L., Yan S. (2017). Robust neighborhood preserving projection by nuclear/L2, 1-norm regularization for image feature extraction. *IEEE Transactions on Image Processing*.

[8] Ginsburg S. B., Lee G., Ali S., Madabhushi A. (2016). Feature importance in nonlinear embeddings (FINE): applications in digital pathology. *IEEE Transactions on Medical Imaging*.

[9] Chen Z., Zou B., Huang M. (2012). Influence of intensity feature on ROI extraction. *Journal of Central South University (Science and Technology)*.

[10] Beck A. H., Sangoi A. R., Leung S. (2011). Imaging: systematic analysis of breast cancer morphology uncovers stromal features associated with survival. *Science Translational Medicine*.

[11] Li K., Yin J., Lu Z. Multiclass boosting SVM using different texture features in HEp-2 cell staining pattern classification.

[12] Di Cataldo S., Bottino A., Ficarra E. Applying textural features to the classification of HEp-2 cell patterns in IIF images.

[13] Al-Kadi O. S. (2010). Texture measures combination for improved meningioma classification of histopathological images. *Pattern Recognition*.

[14] Rodriguez-Galiano V. F., Ghimire B., Rogan J., Chica-Olmo M., Rigol-Sanchez J. P. (2012). An assessment of the effectiveness of a random forest classifier for land-cover classification. *ISPRS Journal of Photogrammetry and Remote Sensing*.

[15] Huang G. B., Ding X., Zhou H. (2010). Optimization method based extreme learning machine for classification. *Neurocomputing*.

[16] Xie Z., Xu K., Liu L., Xiong Y. (2014). 3D shape segmentation and labeling via extreme learning machine. *Computer Graphics Forum*.

[17] Huang G.-B., Zhou H., Ding X., Zhang R. (2012). Extreme learning machine for regression and multiclass classification. *IEEE Transactions on Systems, Man, and Cybernetics B: Cybernetics*.

[18] Zarella D M., Breen E D., Plagov A. (2015). An optimized color transformation for the analysis of digital images of hematoxylin &amp; eosin stained slides. *Journal of Pathology Informatics*.

[19] Sebastian V., Unnikrishnan A., Balakrishnan K. (2012). *Gray level co-occurrence matrices: Generalisation and some new features*.

[20] Zhao Y., Jia W., Hu R.-X., Min H. (2013). Completed robust local binary pattern for texture classification. *Neurocomputing*.

[21] Lindeberg T. (2012). Scale invariant feature transform. *Scholarpedia*.

[22] Jing J., Zhang H., Wang J., Li P., Jia J. (2013). Fabric defect detection using Gabor filters and defect classification based on LBP and Tamura method. *Journal of the Textile Institute*.

[23] Novaković J. (2016). Toward optimal feature selection using ranking methods and classification algorithms. *Yugoslav Journal of Operations Research*.

[24] Chandrashekar G., Sahin F. (2014). A survey on feature selection methods. *Computers & Electrical Engineering*.

[25] Begum S., Bera S. P., Chakraborty D., et al Breast cancer detection using feature selection and active learning.

[26] Gu S., Cheng R., Jin Y. (2016). Feature selection for high-dimensional classification using a competitive swarm optimizer. *Soft Computing*.

[27] Ngiam J., Chen Z., Bhaskar A. S. (2011). Sparse filtering. *Advances in Neural Information Processing Systems*.

[28] Irshad H., Veillard A., Roux L., Racoceanu D. (2014). Methods for nuclei detection, segmentation, and classification in digital histopathology: A review-current status and future potential. *IEEE Reviews in Biomedical Engineering*.

